# Classification of High-Activity Tiagabine Analogs by Binary QSAR Modeling

**DOI:** 10.1002/minf.201300020

**Published:** 2013-05-15

**Authors:** Andreas Jurik, Regina Reicherstorfer, Barbara Zdrazil, Gerhard F Ecker

**Affiliations:** [a] University of Vienna, Department of Medicinal ChemistryAlthanstraße 14, A-1090 Vienna, Austria phone/fax: +431-4277-55110/-9551

**Keywords:** GAT-1, Binary QSAR, Tiagabine, GABA uptake inhibitors

Termination of GABA-ergic signaling requires fast uptake of the neurotransmitter by highly selective transporter proteins. Four subtypes of sodium- and chloride-dependent GABA transporters exist, GAT-1 being the most prominent one in the brain. The only marketed drug targeting this transporter system is the anticonvulsant tiagabine.^1^ It is highly GAT-1 selective, consisting of *R*-nipecotic acid as a GABA mimetic moiety and a diaryl region attached by a linker chain.^2^–^3^ Its development roots back to the work of Yunger et al. in the early 1980s, introducing a lipophilic diaromatic region to the amino acid, thus tackling the fact that nipecotic acid, already a potent inhibitor of GABA transport, is not able to penetrate the blood brain barrier.^4^ This resulted in the so-called SK&F tool compounds, which were subsequently optimized towards *IC*_50_ values in the nanomolar range and simultaneously rising GAT-1 selectivity. Lots of synthetic effort focusing on modifications in linker length and polarity, and substitutions on the (mainly di-) aromatic region has been put into the structural optimization of the compound class, as summarized by Madsen et al.^5^ Modifying the amino acid region is less tolerated, but might be the key for stepping towards other GAT subtypes. Likewise, the introduction of a third aryl ring goes along with an increase in selectivity for hGAT-3.^6^–^7^ It also turned out that *ortho*-substitution of at least one of the aromatic rings has a beneficial effect. In addition, introduction of a polar region at the distal side of the aliphatic linker, which is connected to the cyclic amino acid at its protonable nitrogen atom, increases activity. This is usually achieved by introducing a diaryloxime or a diarylvinyl ether group. Isolated investigation of the preferred carboxy group configuration in this GABA mimetic moiety showed a clear superiority of *R*-configuration to the non-racemic guvacine scaffold, itself being more potent than compounds containing *S*-nipecotic acid.^8^,^9^ Despite the considerable number of structure-activity relationship observations that have been described,^6^,^10^ a quantitative summary of their respective contributions has not been performed yet.1

**Figure 1:**
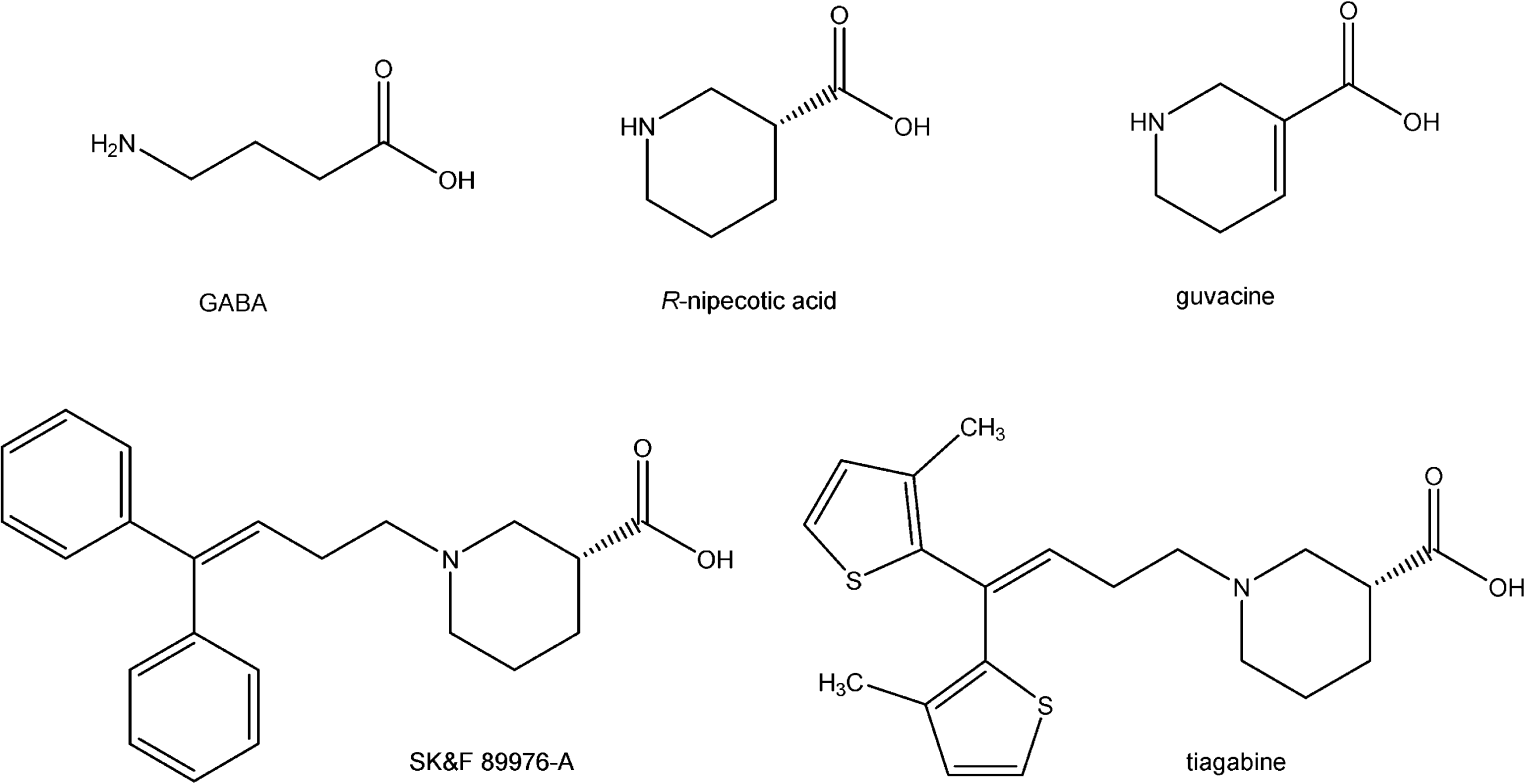
Chemical structures of GABA, *R*-nipecotic acid, the lipophilic derivatives SK&F 89976-A and tiagabine.

In the present work, we describe a ligand-based approach to summarize SAR information derived from a dataset of published lipophilic aromatic GAT inhibitors.

A dataset of 162 consistently tested compounds was collected from the literature.^8^,^9^,^11^–^19^ Two classes of 2D and internal 3D descriptors were calculated using the software package MOE2012.10.^20^ The 2D class, not depending on the molecule conformation, consisted of 188 descriptors belonging to 7 categories, namely physicochemical properties, subdivided surface area, atom and bond counts, Kier & Hall connectivity and kappa shape Index, adjacency and distance matrix, pharmacophore feature and partial charge descriptors.

Out of the available 3D descriptors, the ′x3D′-class was discarded as it depends on external coordinates as a frame of reference. The remaining ′i3D′ class consisted of 138 features, describing shape, potential energy and partial charges of the dataset. In addition, indicator variables were introduced for the three scaffolds of the amino acid mimicry, namely *R*- and *S*-nipecotic acid and guvacine. Three columns were added to the dataset, one for each scaffold. Presence or absence of the respective scaffold in the chemical structure was indicated by 1 and 0, respectively. The full data matrix is given in the supplementary material. Surprisingly, although the data set seems ideal for Hansch-anaylsis and PLS, all attempts to retrieve statistically significant models failed. Therefore, the strategy was adjusted towards binary QSAR. The according method implemented in the QuaSAR module of MOE2012 uses a biased Bayesian inference technique in order to predict the probability of a compound to be active or inactive, even for small and unbalanced data sets.^21^

A p*IC*_50_ activity threshold of 7.0 was defined for discrimination between highly active and inactive compounds. Four different descriptor sets were used for building the binary models: 16 physicochemical descriptors, 32 binned VSA descriptors plus the three indicator variables, one set of features chosen by contingency analysis (see Table 1), and the first 10 principal components of the internal 3D descriptors. In order to assess the quality of the models, internal validation by leave-one-out cross-validation and prediction of an external test set was performed. For the latter, two procedures were applied to split the 162 compounds into 147 (90 %) for training and 16 (10 %) for testing. In order to achieve maximum diversity in the test set, primary splitting of the compounds was done on the basis of maximum diversity, calculated by MACCS fingerprint clustering. The second method used ten times repeated random selection. Upon selection of the test set compounds, it was also taken care of preserving the ratio between active and inactive compounds in both subset populations.

Using the whole panel of 2D-descriptors followed by backward selection as well as the sole use of the 16 physicochemical descriptors did not provide any reasonably good model. Also 3D descriptors performed poorly and were thus discarded. Nevertheless, a set of 32 binned van der Waals surface area (VSA) descriptors turned out to be well suited to describe the dataset. Introducing the indicator variables outlined above increased both positive and negative predictive power for the external test set from 42.9 % and 77.8 % to 60.0 % and 81.8 %, respectively, clearly justifying their use (Table 2). The second feature selection method applied used the descriptor contingency calculation available in the MOE package (Table 1). For the diversity split, it were 9 mainly atom/bond count, adjacency matrix and polarity descriptors, performing equally well when compared to the VSA descriptors for the training set, but exhibiting inferior positive predictive power for the test set (Table 2).

**Table 1:** Descriptors chosen by contingency analysis for the two training sets and their (mean) relative importance

Descriptor	Description	Rel. importance
	**Maximum diversity split**	
a_count	Number of atoms (incl. implicit H)	0.283
b_1rotN	Number of rotatable single bonds	0.235
b_1rotR	Fraction of rotatable single bonds	0.236
b_count	Number of bonds (incl. implicit H)	0.274
b_rotN	Number of rotatable bonds	0.233
b_rotR	Fraction of rotatable bonds	0.250
PEOE_VSA_FPPOS	Fractional positive polar van der Waals surface area	0.218
vdw_area	Area of van der Waals surface (Å^2^)	0.283
wienerPol	Wiener polarity number^22^	0.240
	**Random splits**	
a_count		0.202
b_count		0.212
b_single	Number of single bonds (incl. H)	0.226
opr_brigid	Number of rigid bonds^23^	0.292
wienerPol		0.293

**Table 2:** Accuracy of the binary QSAR models for training and test sets (%)

	A^[a]^	A0^[b]^	A1^[c]^	XA^[d]^	XA0	XA1
**Maximum diversity split**						
*Training set*						
VSA+ind. var^[e]^	89.7	85.1	98.10	85.6	83.0	90.4
2D contingency^[f]^	86.3	88.3	82.7	80.1	84.0	73.1
*External test set*				MCC		
VSA+ind. var	75.0	81.8	60.0	0.42		
2D contingency	75.0	90.9	40.0	0.37		
**Random splits**						
*Training set*						
VSA+ind. var	91.3	88.2	97.1	86.8	86.1	88.2
2D contingency	86.1	86.6	85.1	80.9	84.4	76.7
*External test set*				MCC		
VSA+ind. var	67.5	80.0	46.7	0.30		
2D contingency	73.8	79.0	65.0	0.46		

[a] Overall accuracy; [b] overall accuracy on inactives=specificity; [c] overall accuracy on actives=sensitivity; [d] accuracy for leave one out (LOO) cross-validation; [e] 32 binned VSA descriptors plus indicator variables;^24^–^25^ [f] set of descriptors selected by contingency calculation.

For the diversity splits, the best model showed an overall accuracy on the training set of 89.7 %, with 98.1 % for active and 85.1 % for inactive compounds. The external test set was predicted with an overall accuracy of 75.0 % (60 % on actives and 81.8 % on inactives), as summarized in Table 2. Accordingly generated models for the ten random splits achieved similar values, performing slightly better on the training sets but exposing lower accuracy for identifying the active instances of the external test sets.

Analyzing the misclassified compounds revealed several insights. VSA descriptors exhibited some difficulties in handling molecules with asymmetrical biaromatic moieties, which are often classified as false positives. Main challenges for 3D descriptors included long linker compounds, large tricyclic moieties and S-configuration of the carboxy group. Just two compounds of the dataset, which is provided in the Supporting Information, were misclassified by at least two models: Cpd. **100**, was the only active one bearing a 7 heavy atom long linker. The other, cpd. **37**, often was assigned to the active class due to its favorable combination of *ortho*-substitution and an oxime moiety in the linker, yet having an *S*-configured carboxy group. Nevertheless, in both cases the p*IC*_50_ value was close to the threshold of 7.0 (see Figure 2 for comparison with the most active compound **69**).

**Figure 2:**
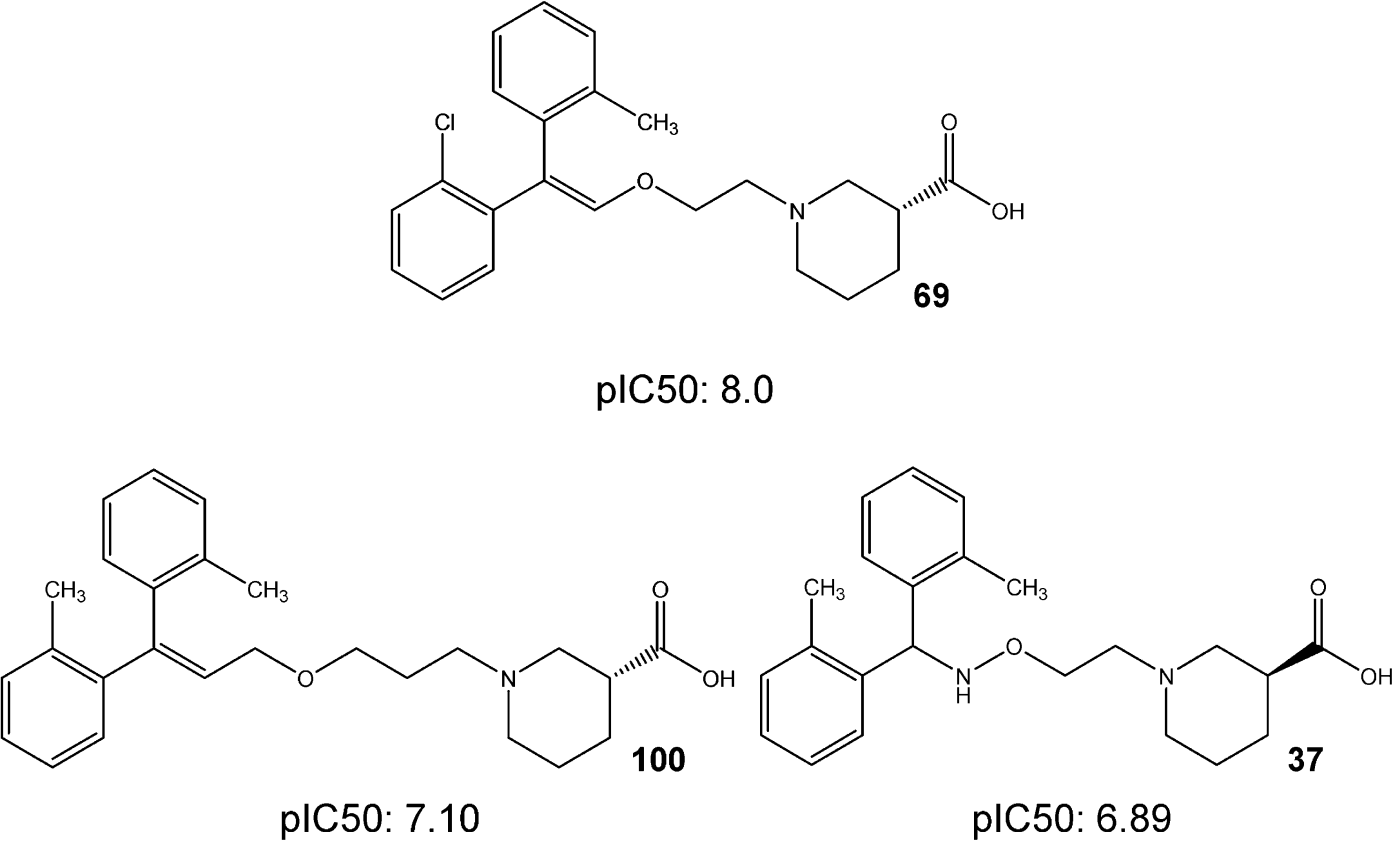
Comparison of the most active compound with most often misclassified compounds. For the most active compound **69**, the optimal linker length and polarity, *ortho*-substitution and *R*-configuration of the carboxy group are present. Frequent false negative cpd. **100** comprises an unusually long linker for active compounds; Cpd. **37** has features typical for highly actives, but lacks the correct stereo configuration.

The surprisingly low importance of the indicator variables during the model generation of the training sets might be explained by the underrepresentation of *S*-configured representatives in the dataset.

New insights about activity-determining features of GABA uptake come from the importance of the two descriptors wienerPol and opr_brigid. Suggested along with three other descriptors in the contingency selection for the random split training set, they perform surprisingly well even if taken alone. Taking just the two for model generation yields Matthews correlation coefficients of 0.53 and 0.63 for training and test set, respectively. Even though the performance is by far weaker when applied to the diverse split, this nicely demonstrates that the degree of rigidity and the polarity distribution play a significantly larger role for activity than expected so far. In contrast, taking just the indicator variables for model generation did not lead to any significant model. In conclusion, BQSAR is a versatile method for capturing SAR information from consistent datasets, when classical QSAR models do not yield sufficient predictive power.

## Computational Methods

*Database preparation.* The initial dataset consisted of more than 400 compounds that were collected from the literature.^4^,^8^–^9^,^11^–^16^,^18^–^19^,^26^ The dataset was curated by removing data from racemic compounds and non comparable inhibition assays, resulting in a final 162 member dataset.

*Threshold.* For the binary QSAR, the compounds were categorized as active according to a p*IC*_50_ of equal to or greater than 7.0, corresponding to 100 nM inhibition of ^3^H-GABA uptake. Any other members of the dataset below the threshold were defined as inactive or 0, dividing the dataset into 57 active and 105 inactive molecules. Compounds annotated with ′larger-than′ *IC*_50_ values were kept in the dataset, as they were all considerably in the micromolar range, thus set inactive at all eligible thresholds.

*Protonation states*. Hydrogen atoms were assigned for the major microspecies at a physiological pH of 7.4 in the target tissue, as well as in the test assay.^27^,^28^ The automatically generated states were cross-checked by random selection of compounds for external p*K*_A_ calculation. Chemicalize.org was used for prediction of p*K*_A_ values and the major microspecies at pH 7.4 (XII 2012; chemaxon.com). Subsequently, a fast energy minimization step was applied in order to ensure reasonable bond lengths, while keeping pre-assigned chiral centers. Further optimized structures were calculated using CORINA.^29^

*Descriptors.* 188 2D descriptors, consisting of 7 categories, as well as 138 internal 3D descriptors of 5 categories, suitable for small molecules, were calculated within the Molecular Operating Environment software package (MOE 2012.10)^20^. For the 2D descriptors, those were 16 physicochemical properties, 18 subdivided surface area, 41 atom and bond counts, 16 Kier & Hall connectivity and kappa shape indices, 34 adjacency and distance matrix, 13 pharmacophore feature and 50 partial charge descriptors. In addition, indicator variables were introduced to describe the amino acid moiety, namely *R*- and *S*-nipecotic acid and guvacine. Out of those, three of the descriptor sets were assembled. Together with the indicator variables, the 18 subdivided surface area descriptors and 14 binned VSA partial charge features shaped the ′VSA′ descriptor set. One consisted of the 16 physicochemical components, as for the third set, all aforementioned features where offered to be chosen by the contingency analysis module within MOE. The internal 3D class consisted of 11 potential energy, 21 MOPAC, 91 surface area, volume and shape features, and 15 conformation dependent charge descriptors; their first ten principal components being used for the fourth model generation approach.

*Selection of training and test set.* Two procedures were applied for dividing the compounds into training and test set prior to model generation.

**a.** The compounds were clustered according to their similarity, determined by calculating MACCS fingerprints at a threshold of 75 % Tanimoto similarity using the Fingerprint Clustering module in MOE2012. As it can be seen in Supporting Information, Scheme 1, clustering at this threshold yields reasonable cluster sizes without over-scattering the dataset. Beginning with a cluster size of minimum 5 members, 1 compound or 10 % were assigned to the test set, chosen according to the actives-inactives distribution within the cluster, also nicely resembling the overall distribution with an actives to inactives ratio of 5 : 11 compared to 57 : 105 in total. Smaller clusters were automatically added to the training set, similar to a procedure described by Fells et al.^30^ For detailed information on the different clusters, see Supporting Information, Table 1.

**b.** The second splitting method was repeated random selection of 6 out of the 57 actives, and 10 out of 105 inactive compounds, representing 10 % of the primary database, yielding ten independent training and test set splits. Detailed description of standard deviation, Matthews correlation coefficient and predictive power of the given mean values can be found in the Supporting Information.

*Contingency analysis.* For both training sets, the calculated descriptors were analyzed for importance and mutual dependence. Four statistical parameters, namely the contingency coefficient, Cramer′s *V*, the uncertainty coefficient *U* and the correlation coefficient *R*^2^, were combined by the contingency module of MOE2012, suggesting a set of descriptors for QSAR models.

*Model generation.* Binary QSAR models were generated within MOE2012, setting a p*IC*_50_ value of 7.0 or above as activity criterion, and adjusting the smooth value to 0.01. Component and condition limit, as well as the binary threshold, were kept at default values.

*Performance measurement.* The models generated for the two training sets were validated by leave one out cross-validation. Both for training and test sets, confusion matrices were drawn, depicting true positives (TP), false positives (FP), false negatives (FN) and true negatives (TN). Overall accuracy (A), accuracy on actives (A1,=sensitivity), accuracy on inactives (A0,=specificity), positive and negative predictive power (PPP, NPP) as well as Matthews correlation coefficient (MCC) were determined as followed:


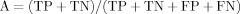



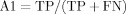



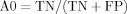



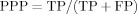



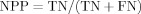



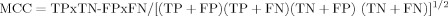


## Supplementary material

As a service to our authors and readers, this journal provides supporting information supplied by the authors. Such materials are peer reviewed and may be re-organized for online delivery, but are not copy-edited or typeset. Technical support issues arising from supporting information (other than missing files) should be addressed to the authors.
