# Supplementary material for: Classification of High-Activity Tiagabine Analogs by Binary QSAR Modeling
Source: Mol Inform. 2013 May 15;32(5-6):415–9. doi: 10.1002/minf.201300020 (PMC3743161; doi:10.1002/minf.201300020)
Supplement: Supplementary file 1 [file minf0032-0415-SD1.pdf]

molecular informatics

models – molecules – systems

Supporting Information

© Copyright Wiley-VCH Verlag GmbH & Co. KGaA, 69451 Weinheim, 2013

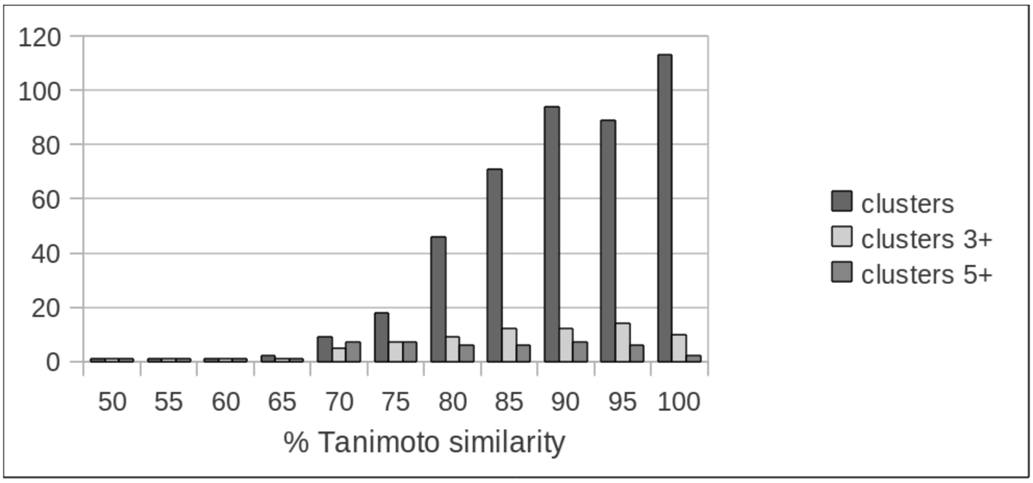

Supporting scheme 1. Similarity thresholds for diversity clustering.

Supporting table 1. Split scheme for training and test sets based on maximum diversity clustering

Cluster	**Training set**		**Test set**		
	Cpds	Split (1/0)	Cpds	Split (1/0)	∑
1	59	25/34	7	3/4	28/38
2	2	2/0			2/0
3	44	18/26	5	2/3	20/29
4	11	3/8	1	0/1	3/9
5	1	1/0			1/0
6[a]	5	2/3			2/3
7	1	1/0			1/0
8	1	0/1			0/1
9	4	0/4	1	0/1	0/5
10	1	0/1			0/1
11	1	0/1			0/1
12	4	0/4	1	0/1	0/5
13	6	0/6	1	0/1	0/7
14	1	0/1			0/1
15	1	0/1			0/1
16	2	0/2			0/2
17	1	0/1			0/1
18	1	0/1			0/1
					**162**

[a] Not considered for test set due to poor activity distribution.

Supporting table 2. Statistical parameters of the repetitive random splits

	A[a]	A0[b]	A1[c]	MCC[d]	PPP[e]	NPP[f]
**Binned VSA descriptors & indicator variables**						
Training set						
Seed 0	0.911	0.863	1.000	0.83	0.797	1.000
Seed 1	0.911	0.863	1.000	0.83	0.797	1.000
Seed 2	0.932	0.926	0.941	0.85	0.873	0.967
Seed 3	0.904	0.863	0.980	0.81	0.794	0.988
Seed 4	0.925	0.905	0.961	0.84	0.845	0.977
Seed 5	0.904	0.853	1.000	0.82	0.785	1.000
Seed 6	0.904	0.884	0.941	0.80	0.814	0.966
Seed 7	0.918	0.916	0.922	0.82	0.855	0.956
Seed 8	0.925	0.895	0.980	0.85	0.833	0.988
Seed 9	0.897	0.853	0.980	0.80	0.781	0.988
SD[g]	*0.011*	*0.027*	*0.028*	*0.019*	*0.032*	*0.016*
External test set						
Seed 0	0.625	0.900	0.167	0.10	0.500	0.643
Seed 1	0.750	0.700	0.833	0.52	0.625	0.875
Seed 2	0.750	0.800	0.667	0.47	0.667	0.800
Seed 3	0.563	0.600	0.500	0.10	0.429	0.667
Seed 4	0.563	0.600	0.500	0.10	0.429	0.667
Seed 5	0.625	0.700	0.500	0.20	0.500	0.700
Seed 6	0.563	0.800	0.167	-0.04	0.333	0.615
Seed 7	0.813	1.000	0.500	0.62	1.000	0.769
Seed 8	0.813	1.000	0.500	0.62	1.000	0.769
Seed 9	0.688	0.900	0.333	0.29	0.667	0.692
SD	*0.101*	*0.149*	*0.205*	*0.242*	*0.230*	*0.081*
**Contingency descriptors**						
Training set						
Seed 0	0.822	0.789	0.882	85.6	83.0	90.4
Seed 1	0.870	0.874	0.863	85.6	83.0	90.4
Seed 2	0.897	0.905	0.882	85.6	83.0	90.4
Seed 3	0.863	0.874	0.843	85.6	83.0	90.4
Seed 4	0.863	0.874	0.843	85.6	83.0	90.4
Seed 5	0.870	0.884	0.843	85.6	83.0	90.4
Seed 6	0.822	0.811	0.843	85.6	83.0	90.4
Seed 7	0.849	0.874	0.804	85.6	83.0	90.4
Seed 8	0.884	0.905	0.843	85.6	83.0	90.4
Seed 9	0.870	0.874	0.863	85.6	83.0	90.4
SD	*0.024*	*0.037*	*0.023*	*0.044*	*0.045*	*0.012*
External test set						
Seed 0	0.813	0.900	0.667	0.59	0.800	0.818
Seed 1	0.688	0.600	0.833	0.42	0.556	0.857
Seed 2	0.688	0.700	0.667	0.36	0.571	0.778
Seed 3	0.750	0.800	0.667	0.47	0.667	0.800
Seed 4	0.813	1.000	0.500	0.62	1.000	0.769
Seed 5	0.750	0.700	0.833	0.52	0.625	0.875
Seed 6	0.563	0.600	0.500	0.10	0.429	0.667
Seed 7	0.813	1.000	0.500	0.62	1.000	0.769

Seed 8	0.688	0.700	0.667	0.36	0.571	0.778
Seed 9	0.813	0.900	0.667	0.59	0.800	0.818
SD	*0.082*	*0.152*	*0.123*	*0.164*	*0.193*	*0.057*

[a] Overall accuracy = (TP+TN)/(TP+TN+FP+FN)

[b] Overall accuracy on inactives (Specificity) = TP/(TP+FN)

[c] Overall accuracy on actives (Sensitivity) = TN/(TN+FP)

[d] Matthews correlation coefficient = TPxTN-FPxFN/$[(TP+FP)(TP+FN)(TN+FP)(TN+FN)]^{1/2}$

[e] Positive predictive power = TP/(TP+FP)

[f] Negative predictive power = TN/(TN+FN)

[g] Standard deviation
